# Supplementary material for: The causal relationship between the human gut microbiota and pyogenic arthritis: a Mendelian randomization study
Source: Front Cell Infect Microbiol. 2024 Nov 26;14:1452480. doi: 10.3389/fcimb.2024.1452480 (PMC11629706; doi:10.3389/fcimb.2024.1452480)
Supplement: Supplementary file 1 [file DataSheet1.zip › mendelian test/ebi-a-GCST90027834.csv_leave_one_out.pdf]

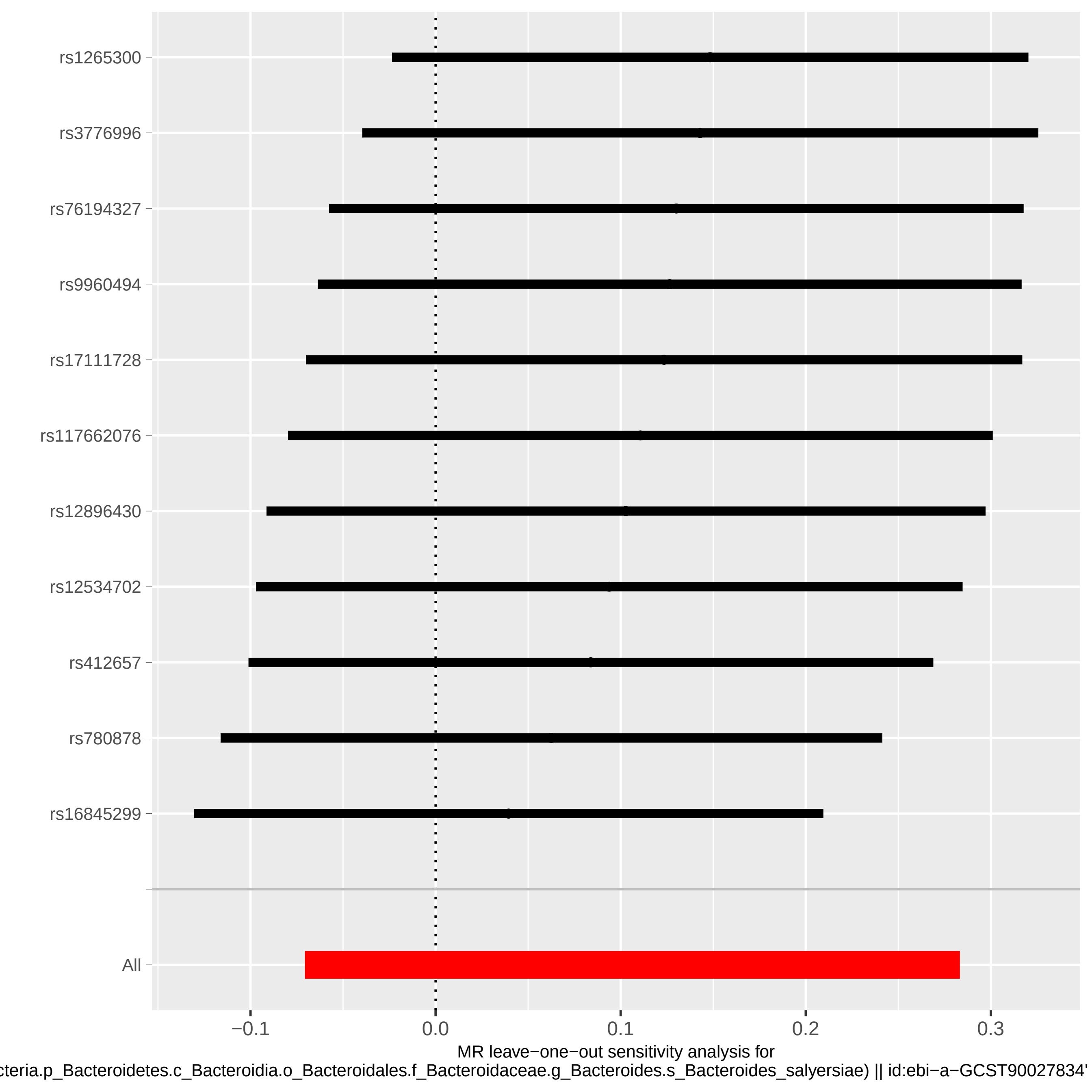
rs1265300
rs3776996
rs76194327
rs9960494
rs17111728
rs117662076
rs12896430
rs12534702
rs412657
rs780878
rs16845299
All
−0.1
0.0
0.1
0.2
0.3
MR leave−one−out sensitivity analysis for
cteria.p_Bacteroidetes.c_Bacteroidia.o_Bacteroidales.f_Bacteroidaceae.g_Bacteroides.s_Bacteroides_salyersiae) || id:ebi−a−GCST90027834'
